# Human Bocavirus in Children with Acute Gastroenteritis, Chile, 1985–2010

**DOI:** 10.3201/eid1911.130601

**Published:** 2013-11

**Authors:** Jorge Levican, Esteban Navas, Joaquín Orizola, Luis Fidel Avendaño, Aldo Gaggero

**Affiliations:** Universidad de Chile, Santiago, Chile

**Keywords:** acute gastroenteritis, human bocavirus, children, viruses, Chile

## Abstract

We detected human bocavirus in 89 (19.3%) of 462 fecal samples collected during 3 periods from 1985 through 2010 from children <5 years of age in Chile who were hospitalized with acute gastroenteritis. Our findings confirm the long-term circulation of human bocavirus in Chile.

Human bocavirus (HBoV) was discovered in 2005 on the basis of large-scale molecular virus screening of respiratory samples (*1*). More recently, HBoV was detected in fecal samples of children who had gastroenteritis with or without symptoms of respiratory infection and in samples from healthy controls (*2*–*4*). Although HBoV is assumed to have coexisted with humans for a long time, there is little evidence to confirm long-term circulation.

## The Study

We analyzed 462 fecal specimens from hospitalized children 0–60 months of age (median 13.8 months) with acute gastroenteritis in Chile. The samples belonged to a collection obtained from 1985 through 2010. Three periods were analyzed: 1985–1986 (period A, 86 samples), 1997–2004 (period B, 261 samples), and 2009–2010 (period C, 115 samples) (Table). The patients did not show respiratory symptoms during their clinical evaluation. Analysis for rotavirus, calicivirus, enteric adenovirus, and astrovirus was conducted (data not shown), and only samples negative for these viruses were selected. The samples were maintained at −80°C until analysis.

**Table:** HBoV in fecal samples from children with acute gastroenteritis, Chile, 1985–2010*

**Period, year**	**Total samples analyzed**	**Samples, no. (%)**			
		HBoV1	HBoV2	HBoV3	Total positive
**A**					
** 1985**	69	12 (17.4)	2 (2.9)	2 (2.9)	16 (23.2)
** 1986**	17	3 (17.7)	0	0	3 (17.7)
** Total**	86	15 (17.4)	2 (2.3)	2 (2.3)	19 (22.1)
**B**					
** 1997**	25	2 (8.0)	1 (4.0)	0	3 (12)
** 1998**	45	6 (13.3)	1 (2.2)	0	7 (15.6)
** 1999**	35	7 (20.0)	1 (2.9)	0	8 (22.9)
** 2000**	31	4 (12.9)	0	1 (3.2)	5 (16.1)
** 2001**	31	7 (22.6)	1 (3.2)	1 (3.2)	9 (29)
** 2002**	31	3 (9.7)	2 (6.5)	1 (3.2)	6 (19.4)
** 2003**	32	6 (18.8)	0	0	6 (18.8)
** 2004**	31	7 (22.6)	3 (9.7)	1 (3.2)	11 (35.5)
** Total **	261	42 (16.1)	9 (3.5)	4 (1.5)	55 (21.1)
**C**					
** 2009**	52	4 (7,7)	4 (7,7)	0	8 (15,4)
** 2010**	63	4 (6,4)	3 (4,8)	0	7 (11,1)
** Total**	115	8 (7)	7 (6,1)	0	15 (13,0)
**Total**	462	65 (14,1)	18 (3,9)	6 (1,3)	89 (19,3)
*HBoV, human bocavirus.					

DNA from fecal samples was extracted by using a High Pure Nucleic Acid Viral Kit (Roche Diagnostics, Indianapolis, IN, USA) following the manufacturer’s instructions. Using PCR with specific primers as described, we performed the HBoV detection (*3*–*5*). Positive and negative controls were included in each amplification round. PCR products were purified, and nucleotide sequences were determined by Macrogen Inc. (Seoul, South Korea) and submitted to GenBank (accession nos. KC757418–KC757460). 

Phylogenetic relationships between isolates from Chile and GenBank reference strains were studied by using MEGA5 software (*6*). We inferred the evolutionary history using the neighbor-joining method. Bootstrap (1,000 replications) was used to assess the reliability of individual nodes in each phylogenetic tree. Evolutionary distances were computed by using the Kimura 2-parameter method (*6*). Variance analysis of the bocavirus detection frequency was conducted by using the Kruskal-Wallis test (α = 0.05) using Statdisk 12.0.1 software (Marc Triola and Pearson Education, Inc., New York, NY, USA). The Ethics Committee of the Faculty of Medicine, University of Chile approved the study.

The 89 (19.3%) samples positive for HBoV were distributed throughout the study period; 22.1%, 21.1%, and 13.0% for periods A, B, and C, respectively (Table). HBoV1 was the most frequently detected species, with 65 (14.1%) cases, followed by HBoV2 and HBoV3, with 18 (3.9%) and 6 (1.3%), cases, respectively (Table). HBoV4 was not detected.

Twenty-two (of 65 HBoV1) nonstructural (NS) 1 partial coding sequences were obtained, and consistent with previous reports, phylogenetic analysis showed that HBoV1 constitutes a genetically homogeneous entity (*4*,*7*). The nucleotide divergence average for the HBoV1 Chile isolates was 0.7% (range 0%–1.7%). In the phylogenetic tree analysis, 15 of 22 isolates clustered with prototype strain st1 (GenBank accession no. DQ000495), and the remaining 5 clustered with prototype strain st2 (GenBank accession no. DQ000496) (Figure, panel A). There was no temporal clustering of the HBoV1 isolates. Similarly, the phylogenetic analysis of 391 nt from the nucleocapsid 1 region of 5 HBoV3 isolates from Chile revealed an average divergence of 1.2% (range 0%–1.6%), and the phylogenetic tree analysis showed that the isolates grouped into 2 clusters with no evident temporal clustering (Figure, panel B).

**Figure:**
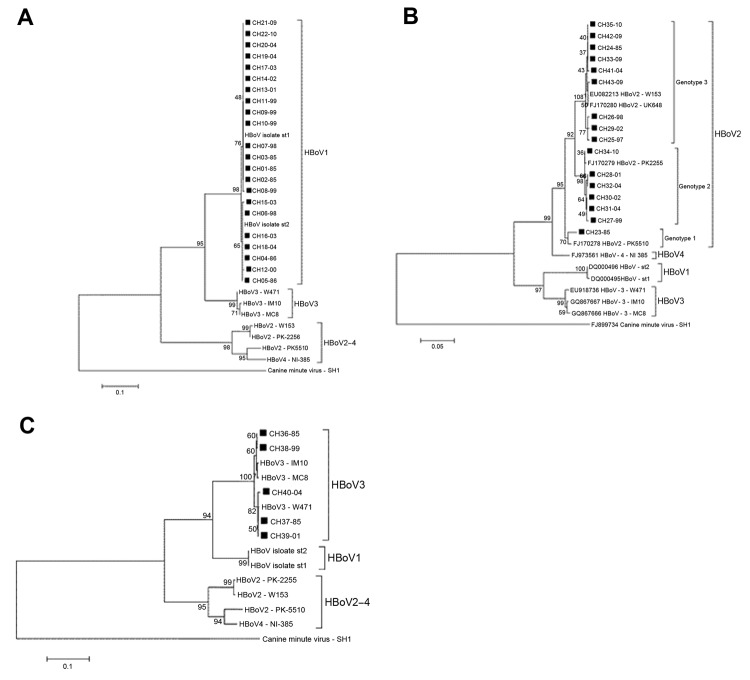
Phylogenetic analysis of nucleotide sequences of isolates of human bocavirus (HBoV), Chile, 1985–2010. A) Phylogenetic analysis of nonstructural (NS) 1 partial region of HBoV1 isolates, positions 554–792, in reference strain HBoV st1 (GenBank accession no. DQ000495). B) Analysis of NS1 partial region of HBoV2 isolates, positions 1427–1881, in reference strain HBoV2 PK225 (GenBank accession no. FJ170279). C) Phylogenetic analysis of nucleocapsid 1 partial region of HBoV3 isolates, positions 2256–2646, in reference strain HBoV3 IM10 (GenBank accession no. GQ867667). Isolates from Chile are indicated by black squares, and the nomenclature used includes sampling years after the dash. Phylogenetic analysis was conducted by using the neighbor-joining method. The reliability of the inferred relations was evaluated by using bootstrap test (1,000 replicates). The evolutionary distances were computed by using Kimura 2-parameter method. The reference strains (GenBank accession numbers) used for phylogenetic analysis were as follows: HBoV st1 (DQ000495), HBoV st2 (DQ000496), HBoV2 PK5510 (FJ170278), HBoV2 PK2255 (FJ170279), HBoV2 W153 (EU082213), HBoV2 UK-648 (FJ170280), HBoV3 W471 (EU918736), HBoV3 IM10 (GQ867667), HBoV3 MC8 (GQ867666), HBoV4 NI385 (FJ973561), and canine minute virus SH1 (FJ899734). Scale bars indicate evolutionary distances used to infer the phylogenetic tree.

In contrast, HBoV2 was a genetically heterogeneous group. Analysis of the nucleotide sequence of the NS1 partial region of 16 of 18 isolates yielded an average of 3.0% nt divergence (range 0%–6.0%). CH23–85, the most divergent isolate, showed 5.0%−6.0% nt divergence with other HBoV2 isolates from Chile. Phylogenetic tree analysis demonstrated that this isolate was closely related to the Pakistan strain PK5510, with which it formed a separate cluster (98% nt identity). This same analysis demonstrated 2 additional clusters among HBoV2 isolates from Chile whose intragroup average nucleotide identity reached 99.6% and 99.5%, respectively.

Kapoor et al. reported a similar clustering pattern by phylogenetic analysis of NS1 and nucleocapsid 1 of HBoV2. They recognized 3 clusters, which enabled them to categorize HBoV2 into 3 genotypes: genotype 1, represented by prototype strain PK5510 (GenBank accession no. FJ170278); genotype 2, with prototype strain PK2255 (GenBank accession no. FJ170279); and genotype 3, with prototype strain UK648 (GenBank accession no. FJ170280) (*4*).

Following this scheme, we determined that 1 of 13 isolates belongs to genotype 1 (CH23–85), 6 to genotype 2 (CH27–99, CH28–01, CH30–02, CH31–04, CH32–04, CH34−10), and 9 to genotype 3 (CH24–85, CH25–97, CH26–98, CH29–02, CH33–09, CH35–10, CH41–04, CH42–09, CH43–09) (Figure, panel C). Unlike HBoV1, which was present in all periods analyzed showing a downward trend in the last period, the different genotypes HBoV2 show a marked dynamism. Thus, period A (1985–1986) revealed only 2 isolates (genotypes 1 and 3).

We found no other isolates of genotype 1 during the remaining study time (Figure, panel C). Genotype 3 became the only genotype prevalent during 1997 and 1998 and remained in the other years studied. Genotype 2 appeared in 1999 and persisted until the last year analyzed; it probably led to increased HBoV2 detection in 2009–10 (Figure, panel C, Table). This prompted speculation that HBoV2 infection is a dynamic phenomenon that can manifest with the emergence of different variants of the agent at different times. This possibility also is rooted in the observation that HBoV2 is highly prone to recombination, and as a consequence presents a high degree of diversity. This diversity is proposed to have given rise to HBoV1, a species with completely different biologic characteristics (*7*). However, because of the lack of continuous chronologic follow-up of the phenomena found in this study, we cannot ensure such assumptions, and a larger longitudinal study is required to confirm this hypothesis.

## Conclusions

Although HBoV1 was originally detected in respiratory secretions of patients with respiratory infection, numerous studies have demonstrated its presence in 1.5%–19% of fecal samples (*1*–*3*,*5**,**8*,*9*). However, after primary respiratory infection this agent can persist with asymptomatic shedding for several months (*10**,**11*). Thus, HBoV1 in fecal samples could be due mainly to passive transfer from the respiratory tract (*8*,*12*).

Unlike HBoV1, HBoV2–4 have enteric tropism, and their role in gastroenteritis remains unclear (*9*). HBoV2 has been detected in feces from children with gastroenteritis in a broad range of percentages (1%–21%) alone or in co-infection with other enteropathogens. In contrast to our study, it has been frequently reported as the main HBoV species detected in feces (*4*,*5*,*9**,**13*). Moreover, in accordance with previous reports that found HBoV3 in low percentages (0%–2%), we detected HBoV3 only in periods A and B (2.3% and 1.5%, respectively) (Table) and did not detect HBoV4 (*5**,**7**,**9**,**14*). Although samples were maintained at −80°C until tested, we cannot exclude the possibility that molecular detection of HBoV may be reduced by long-term storage. This fact, along with the co-infection exclusion, may explain the low detection rates of the strains with enteric tropism.

Although the frequency of detection of HBoV species varied among the periods studied, variance analysis indicated no significant differences (p = 0.099) (Table). We cannot confirm circulation or variation in detection in periods during which surveillance was not conducted.

This study confirms long-term circulation of HBoV in Chile and demonstrates the heterogeneity of HBoV2. These findings justify prospective studies to better understand the role of these viruses in childhood gastroenteritis.
